# Sustainable blue economy: energy consumption and carbon‑neutrality strategies for Albania’s marine and fisheries sectors

**DOI:** 10.1186/s13021-025-00337-w

**Published:** 2025-11-04

**Authors:** Lorenc Malka, Edmond Zeneli, Nadjem Bailek, Evgjeni Xhafaj, Bledar Sakaj, Shabana Urooj, Jihad A. Younis, El-Sayed M. El-Kenawy

**Affiliations:** 1 Energy Department, Mechanical Engineering Faculty, Polytechnic University of Tirana, Tirana, Albania; 2 Department of Mathematics and Computer Science, Laboratory of Mathematics Modeling and Applications, Faculty of Sciences and Technology, Ahmed Draia University of Adrar, Adrar, 01000 Algeria; 3 Department of Mathematical Engineering, Faculty of Mathematical and Physics Engineering Polytechnic University of Tirana, Tirana, Albania; 4 Vlora Port Authority, Albanian Maritime Sector, Vlora, Albania; 5 Department of Electrical Engineering, College of Engineering, Princess Nourah bint Abdulrahman University, P.O. Box 84428, Riyadh, 11671 Saudi Arabia; 6 Department of Mathematics, Aden University, Aden, Yemen; 7 School of ICT, Faculty of Engineering, Design and Information & Communications Technology (EDICT), Bahrain Polytechnic, PO Box 33349, Isa Town, Bahrain; 8 Applied Science Research Center, Applied Science Private University, Amman, Jordan

**Keywords:** EnergyPLAN, Low-Carbon strategies, Carbon neutrality, Sustainable blue economy, Sustainable development, Transport sector, Pollution, Smart energy system, Energy transition, Energy consumption

## Abstract

The maritime transportation and fisheries sectors play a crucial role in national food security and economic stability; however, they face significant sustainability challenges due to climate change and carbon emissions. This study investigates mitigation scenarios for achieving carbon neutrality in the sector by 2050, addressing both energy demand and emissions mitigation in accordance with the IMO 2023 decarbonization targets. Quantitative modeling using the Low Emissions Analysis Platform system, combined with multiple linear regression analysis, is utilized to simulate long-term energy demand, fuel substitution, and emissions across fisheries and navigation sub-sectors. The analysis compares the baseline scenario of continued fossil fuel reliance with an alternative decarbonization scenario. Under the baseline scenario, energy demand and emissions rise sharply, while the alternative scenario projects a 71.1% reduction in fisheries-related emissions and a 76.4% reduction in the navigation sector by 2050. Fossil fuel dependency declines from full reliance in 2025 to 5% in 2050, replaced by a diversified mix of renewable fuels. Total energy demand stabilizes at 0.723 TWh under the decarbonization pathway compared to 0.980 TWh under baseline conditions. Projected hydrogen adoption grows from 10% in 2030 to 30% in 2050, while biodiesel follows a comparable growth curve. Anticipated mitigation of 1.473 MtCO₂eq underscores the sector’s potential to meet national climate targets when underpinned by regulatory support mechanisms and targeted investments. This study underscores the potential for Albania’s maritime sector to lead decarbonization efforts in the Mediterranean region through an integrated strategy combining policy reform, technology adoption, and regional cooperation.

## Introduction

### Overview

The ongoing global energy crisis, fluctuating fuel prices due to geopolitics, the emerging climate change agenda, and continuous geopolitical issues have pushed countries to design strategies to manage energy resources and ensure security of supply over time. These strategies encompass both aspects of the entire energy system: the demand side (households, transport, industry, services, agriculture, fisheries, and others) and the supply side, which is responsible for energy generation to meet overall energy demands [1]. The total global primary energy consumption is growing continuously to around 620 EJ by the end of 2023, which means an increase of 40% higher compared to the 2000 level [2]. Within this context, the transportation sector is responsible for consuming around a quarter of total world demand and contributing to around 25–30% of total emissions worldwide, while international shipping is responsible for 2.8–3% of all global GHG emissions [3]. Trends show that the sector continues to grapple with how to meet the greenhouse gas (GHG) emission targets as determined by the International Maritime Organization (IMO) [4]. in its 2018 initial strategy on reduction of GHG emissions from ships, as well as the most recent revised strategy (IMO, 2023a, Annex) [5].

The International Maritime Organization (IMO) formalized its Initial Greenhouse Gas (GHG) Reduction Strategy in 2018, establishing a pivotal framework for the mitigation of vessel-derived emissions within the maritime sector. This strategic directive delineated quantifiable decarbonization benchmarks, aiming to curtail the carbon intensity of shipping operations and transition the industry toward low-emission propulsion technologies and alternative energy carriers [4]. The resolution encouraged flag states to take proactive steps to make the shipping sector carbon neutral. The IMO’s 2050 targets will be achieved via a radical technology shift together with the aid of social pressure, financial incentives, and regulatory and legislative reforms at the local, regional, and international levels [6]. Hybrid propulsion systems play a crucial role in advancing low-emission shipping sectors worldwide. Leveraging pattern recognition, an adaptive energy control strategy to optimize power distribution between generators and batteries by using nonlinear predictive control, the system dynamically adjusts energy flow in real time, minimizing fuel consumption and enhancing operational efficiency is proposed [6]. Optimizing vessel speed fields can mitigate carbon emissions in the maritime industry. Focusing on liner shipping routes between China and Europe, the study [3] examines how carbon pricing mechanisms, including carbon taxes and emissions trading under the European Union Emissions Trading Scheme (EU ETS), affect operational costs and emissions reduction levels. Furthermore, with the application of more stringent maritime environmental regulations, hybrid fuel cell ships have garnered significant attention due to their merits in low specific emissions and high efficiency. However, difficulties related to the coordinated control of multi-energy systems and fuel cell degradation remain significant constraints to their practical adoption [7]. For the period between 2017 and 2020, the core shipping decarbonisation technologies were condensed to identify ‘pros and cons’ using the most recent academic literature applications. Furthermore, the review of the trajectory of shipping decarbonisation research themes indicates clear research gaps in the current literature and guides the development of a future research agenda with the prediction of future opportunities and potential for shipping decarbonisation research developments for the shipping industry [8]. The International Maritime Organization (IMO) Fourth GHG Study estimated the current carbon footprint of the shipping industry and projected GHG emissions from shipping. In the study by [9], the interlinkages between transport, energy, industry, agriculture, and forestry, by including synergies and trade-offs to inform more effective and integrated decarbonization strategies are evaluated.

The shipping industry, an anchor of the global economy, is experiencing a radical shift with the introduction of artificial intelligence (AI). This pivotal sector, responsible for transporting over 90% of the world’s trade goods, endures an array of difficulties, including operational efficiency, risk mitigation, environmental sustainability, and supervisory guidelines. Through AI, the shipping industry is noting significant transformative progress in predictive maintenance, autonomous operations, route optimization, load processing and handling, and strict environmental management. Predictive analytics enables shipping companies to refine, with precision, vessel scheduling, enhancing efficiency, reducing delays, and optimizing fleet dispatch and data planning such as destination, arrival time, trajectory, and trip duration offered by the port community platforms to manage their trips most efficiently [10]. As of the latest projections, the shipping industry is moving towards digitization and smart technologies, which reflects not just a trend, but a necessary evolution, with the global maritime freight market expected to reach $14 billion, growing at a Compound Annual Growth Rate (CAGR) of 3.2%.

The transport sector in Albania relies totally on fossil resources, except for road transportation, with a very negligible portion of 0.4% of the total road passengers’ fleet, and only 2.3% of new registered vehicles in 2024, while other sub-branches including shipping, rail, and air are fully based on fossil fuel sources as is the fisheries sector. As a result, the transport sector ranks first in terms of consumption and pollution in Albania [11, 12]. The vision of a carbon-free, sustainable, flexible, and environmentally friendly shipping transport system based on RES, and intensifying electrification and biofuels will absolutely shape the future toward a fully decarbonized transport sector in meeting the 2050 roadmap and climate goals in Albania, one of the conditions that will make the country’s integration process into the European Union easier. In this regard, scientific research in the maritime industry, logistics, and shifting to more sustainable energy sources highlights the importance of energy security, environmental and modernizing port facilities by improving energy efficiency as some of the pathways for a safer shipping sub-branch and fishing sector.

Continued investment in port infrastructure and technological advancements will ensure long-term viability and may have a substantial impact on the country’s economy, easily integrable with the road passenger and freight sectors as they share more than 90% of the freight activity in Albania. Thereafter, shifting to cleaner fuels and electrification of the shipping subsector will be the prominent lever of energy consumption used by transport by the end of 2050 [13]. Hence, stimulating the growth of renewable energy technologies within the national energy system will play an instrumental role in the evolution toward a 100% RES-based system, including the transport sector, mitigating the negative human health effects and performing with less energy per activity level.

Implementing new policies and measures toward smart and sustainable transport, including a revision of the current NECP [14] and fulfilling the requirements of Law No. 24/2023 on “Promoting the Use of Energy from Renewable Sources” [15, 16], is very deterministic for further developments of the sector. Besides bio-fuel, other clean options should be selected in compliance with other developments, as given in the study [17], conducting the techno-environmental assessment of methanol production using chemical looping technologies, highlighting the fact that chemical looping technologies reduce greenhouse gas emissions (GWP) by almost 25%.

### Study purpose and objectives

Despite extensive global research on maritime decarbonization and scenario analysis, there is no comprehensive, Albania-specific evaluation that concurrently integrates multiple alternative fuels, techno-economic feasibility, policy frameworks, and energy storage requirements for both shipping and fisheries. Therefore, this study aims to (1) fill that gap by developing and comparing baseline and decarbonization scenarios tailored to Albania’s maritime and fisheries subsectors; (2) quantify long-term energy demand, fuel-mix transitions, and GHG emissions under each scenario; and (3) formulate targeted policy and investment recommendations to support Albania’s pathway to carbon neutrality in the maritime sector by 2050.

## Shipping and fisheries sector overview

Albania’s maritime sector, facilitated by its strategic 446-kilometer Adriatic and Ionian coasts, is the cornerstone of national economic and logistical infrastructure. The industry is dominated by four commercial seaports—Durrës, Vlorë, Shëngjin, and Saranda—collectively facilitating trade, tourism, and industry. Among these, the Port of Durrës plays a predominant role, handling approximately 96% of the country’s maritime cargo and more than 80% of passenger traffic. Secondary ports such as Vlorë and Saranda complement this network by handling niche cargo operations and 11.6% and 7.5% of passenger traffic, respectively.

**Fig. 1:**
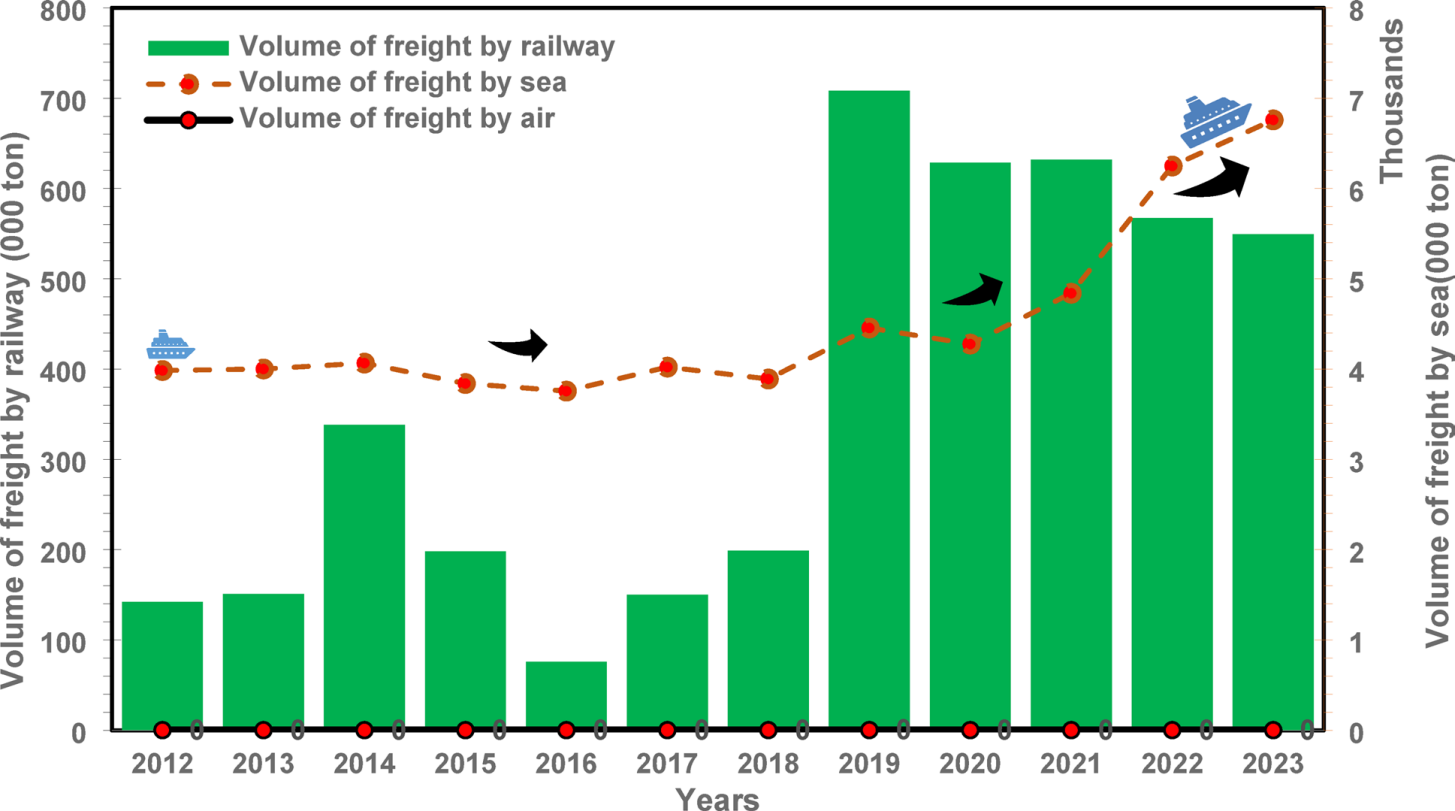
Freight transport by mode (excluding Road): Dynamic trends in railway, sea, and air volumes activity (Thousands of Tonnes), 2012–2023

The transport of freight in Albania from 2012 to 2023, including movements by sea, air, and rail, exhibits a complex interplay of energy use and modal dominance, as illustrated in Fig. 1. Maritime shipping contributes only 4–5% to the total transportation energy demand, amounting to roughly 0.4 TWh annually. However, its performance demonstrates a consistent level of reliability compared to the broader transport sector. During the pandemic-related downturn in 2020, when overall transport energy demand fell from 10.00 TWh in 2019 to 7.29 TWh, the energy consumption in maritime transport remained stable. This stability underscores the sector’s sustained importance in maintaining trade flows during periods of disruption.

**Fig. 2:**
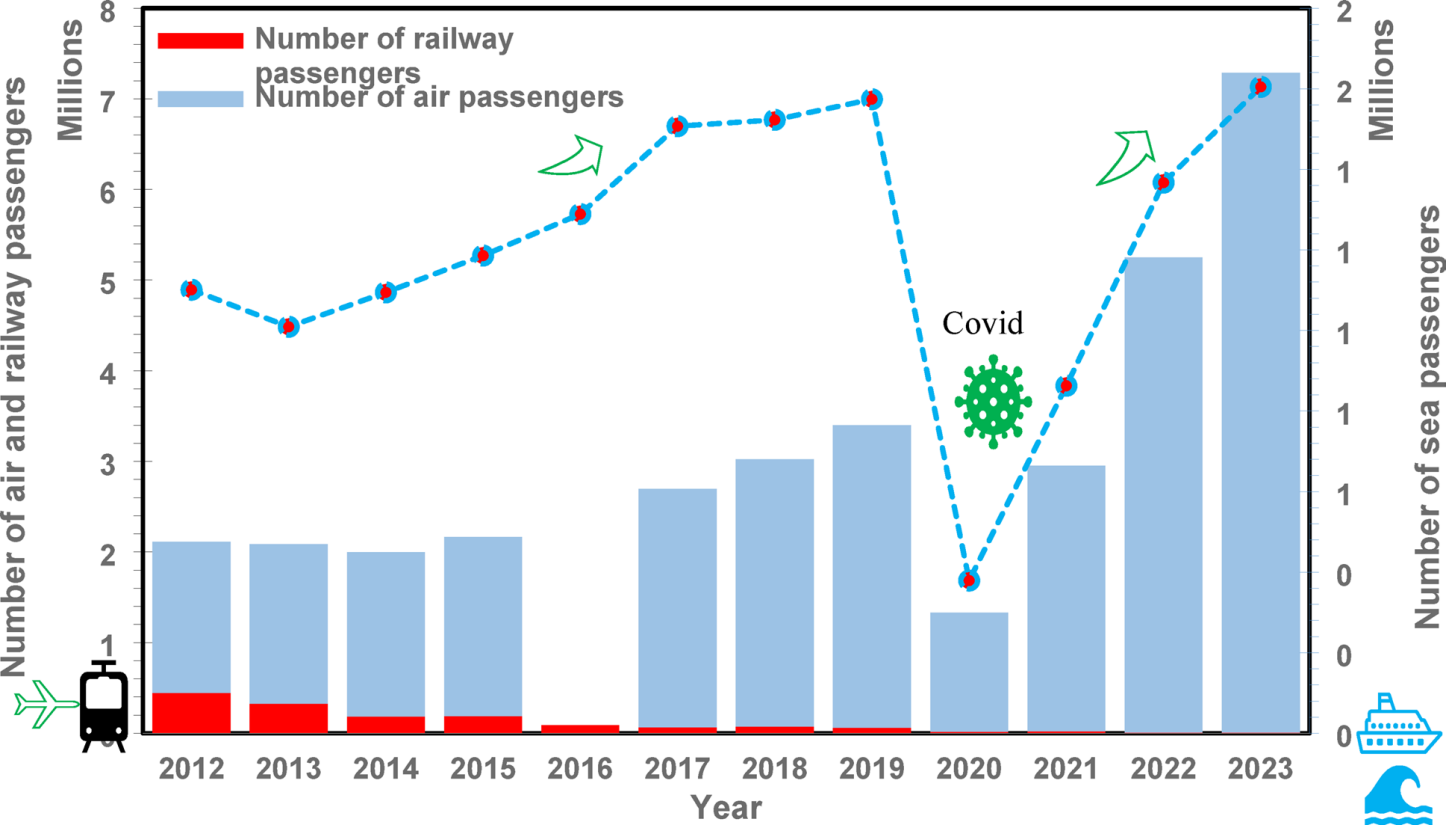
Passenger transport by mode (excluding Road): Trends in railway, maritime, and air passenger activity (Millions of Passengers), 2012–2023

Figure 2 presents the progression of passenger volumes across rail, sea, and air transport within the same period. Rail transport has experienced a persistent and severe decline, with annual passenger numbers dropping from 448,000 in 2012 to only 7,363 in 2023, indicating a functional deterioration of this mode. Sea transport, though subject to intermittent fluctuations, registered moderate growth, increasing from approximately 1.1 million passengers in 2012 to approximately 1.6 million passengers in 2023. The trajectory was temporarily affected by the COVID-19 pandemic, which resulted in a momentary decline. Air travel demonstrated the most substantial expansion, growing from 1.7 million passengers in 2012 to over 7.2 million in 2023.

**Table 1:** A dynamic summary of fishery activity levels and energy consumption over two decades (2005–2024)

Variable	Period	Mean	Median	Std Dev	Min	Max
Activity Level (kMT)	2005–2010	7.2	7.4	0.4	6.4	7.6
Activity Level (kMT)	2011–2015	9.7	11	2.0	7.4	11
Activity Level (kMT)	2015–2024	14.3	14.9	1.6	11	16
Final Energy Intensity (toe/MT)	2005–2010	3.4	3.65	1.0	2	4.5
Final Energy Intensity (toe/MT)	2011–2015	2.9	2.9	0.4	2.4	3.4
Final Energy Intensity (toe/MT)	2015–2023	2.9	3	0.2	2.3	3

Table 1 presents a summary of fisheries activity and energy intensity trends in Albania across three distinct periods. A marked increase in fishery output is evident, with the mean production rising from 7.2 thousand metric tonnes (kMT) during 2005–2010 to 9.7 kMT in 2011–2015, and further to 14.3 kMT in 2015–2024. This represents an approximate doubling of output over two decades. Maximum recorded production values also followed a similar upward trajectory, increasing from 7.6 kMT to 16 kMT across the same periods. Production volatility, measured by standard deviation, rose from 0.4 kMT in 2005–2010 to 2.0 kMT in 2011–2015, before declining to 1.6 kMT in 2015–2024, indicating greater stability in recent years.

In parallel, improvements in energy efficiency were observed. Mean energy intensity declined from 3.4 toe per metric tonne in 2005–2010 to 2.9 toe/MT in 2011–2015, maintaining this level through 2015–2024. The minimum recorded value increased to 2.3 toe/MT in the latest period, and the standard deviation decreased from 1.0 to 0.2, reflecting more uniform energy use across the sector. However, the persistence of energy intensity levels above 2.3 toe/MT highlights the need for deeper innovation to align with decarbonization targets.

Fishery activity increased by 34.7% from 2005 to 2010 to 2011–2015, and by 47.4% from 2011 to 2015 to 2015–2024, signaling robust sectoral growth. In contrast, energy intensity declined by 14.7% from the first to the second period but remained unchanged thereafter, suggesting a plateau in efficiency gains. The ratio of activity level to energy intensity improved from 2.12 kMT/toe in 2005–2010 to 3.34 kMT/toe in 2011–2015, and further to 4.93 kMT/toe in 2015–2024, indicating higher productivity per unit of energy consumed. Additionally, the ratio of maximum to minimum energy intensity narrowed from 2.25 in the earliest period to 1.42 in the most recent. These trends suggest that the fisheries sector has managed to decouple production growth from energy consumption, but additional technological and management improvements are needed to align with Albania’s decarbonization objectives.

## Methodology

### Activity level prediction method

MLR methodologies are widely used for the modeling of the energy system since they are interpretable, transparent, and versatile across socio-economic contexts [18]. The strength of MLR lies in **its** ability to determine the linear relationship between the predictors and the dependent variable, and therefore it is a useful tool for scenario analyses involving energy [19, 20]. MLR was recently applied **to** the forecasting of industrial demand for energy [22] and domestic demand for electricity [21], attesting to its versatility. MLR is most useful where historic data are limited, a common problem with developing economies such as Albania. With the use of MLR combined with planning models like LEAP, policymakers are able to make data-based plans for emissions mitigation and energy security [22]. The conventional MLR model for the prediction of activity level is given by:

1 $$\:Y={\beta\:}_{0}+\sum_{i=1}^{n}{\beta\:}_{i}{X}_{i}$$

where: $\:Y$ : Forecasted level of activity (e.g., industry production, energy usage); $\:{\beta\:}_{0}$ : Intercept term; $\:{\beta\:}_{i}$ : Coefficients of the predictor variables; $\:{X}_{i}$ : Predictor variables, where $\:{X}_{1}$ represents GDP (in billions of euros), and $\:{X}_{2}$ depicts population trends from the study.

To estimate the coefficients $\:\beta\:$, the Ordinary Least Squares (OLS) method is used, minimizing the squared residuals:

2 $$\:\text{m}\text{i}\text{n}\sum_{j=1}^{m}{\left({Y}_{j}-\left({\beta\:}_{0}+\sum_{i=1}^{n}{\beta\:}_{i}{X}_{ij}\right)\right)}^{2}$$

where: $\:m$ is the number of observations, $\:n$ is the number of predictor variables.

In a quest for better model accuracy, cross-validation techniques and multicollinearity testing (using the Variance Inflation Factor, VIF) are typically used. The adjusted $\:{R}^{2}$ metric determines model fitness:

3 $$\:{R}_{\text{adj}}^{2}=1-\left(\frac{\left(1-{R}^{2}\right)\left(m-1\right)}{\left(m-n-1\right)}\right)$$

where: $\:{R}^{2}$ is the coefficient of determination, which indicates the proportion of the explained variance.

In the current application, the multivariable regression model is trained on 12 years of data (2012–2023), incorporating trends in activity levels, GDP, and population in Albania. The resulting forecast of activity levels, when multiplied by corresponding energy intensity values, yields estimates of energy demand expressed in terawatt-hours [11].

### Evaluating multi-pollutant emissions

Low Emissions Analysis Platform (LEAP) is a widely used energy system modeling software employed for the assessment of emissions abatement measures. LEAP, developed by the Stockholm Environment Institute, is applied for planning energy policy and the mitigation of climate change [23, 24]. The software facilitates the estimation of emissions other than CO₂, such as CH₄, NOₓ, PM₂.₅, and SO₂, and thus provides support for a broader environmental effects assessment [25]. Research in the recent past has shown the application of LEAP for the examination of low-carbon development options as well as control measures for multi-pollutant emissions for various sectors like energy, transportation, and industry [26, 27]. The assessments facilitate the optimization of policy-making as well as ensure sustainable development [28]. However, LEAP has some limitations: it uses a low temporal granularity (typically annual or seasonal rather than hourly), which can obscure intra‑annual variability; it demands extensive input data that may not be available in data‑scarce regions; and it does not include an integrated optimization, so cost‑minimization or multi‑objective optimization must be conducted via external tools.

In a final energy demand analysis, energy demand is computed as the product of the total activity level and energy intensity for each technology branch. The general formula is [29]:

4 $$\:\begin{array}{cccc}&\:{D}_{b,s,t}={\text{TA}}_{b,s,t}\times\:{\text{EI}}_{b,s,t}\end{array}$$

where:

- $$\:{D}_{b,s,t}$$: Energy demand.
- $\:{\text{TA}}_{b,s,t}$: Total activity level.
- $$\:{\text{EI}}_{b,s,t}$$: Energy intensity.
- Subscripts $\:b$, $\:s$, $\:t$: Branch, scenario, and year (base year $\:t=0$).

For the base year ($\:t=0$):

5 $$\:\begin{array}{cccc}&\:{D}_{b,0}={\text{TA}}_{b,0}\times\:{\text{EI}}_{b,0}\end{array}$$

The total activity level $\:{\text{TA}}_{b,s,t}$ is derived by multiplying activity levels across hierarchical branches (e.g., parent $\:{b}^{{\prime\:}}$, grandparent $\:{b}^{{\prime\:}{\prime\:}}$):

6 $$\:\begin{array}{cccc}&\:{\text{TA}}_{b,s,t}=\prod_{k=1}^{K}{A}_{{b}^{\left(k\right)},s,t}\end{array}$$

LEAP is an energy model that accounts for emissions other than CO₂ and allows users to calculate the 100-year GWP (at the emission point) environmental impact, as well as other emissions.

### Scenario conceptualization and assumptions in modelling

**Fig. 3:**
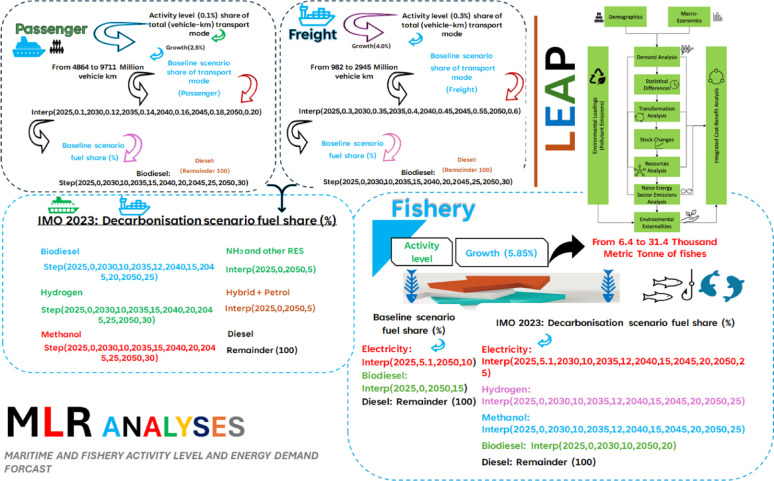
LEAP model structure, adapted after Charles [30, 31]

This section establishes the analytical framework and assumptions for evaluating Albania’s carbon‑neutral fisheries and marine systems path. The methodology is twofold, as illustrated in Fig. 3, through the integration of cost–benefit analysis across multiple dimensions and scenario design. Two scenarios—baseline and IMO 2023 decarbonization (proposed scenario)—are built to systematically evaluate fuel transition pathways, technological adoption, and sectoral resilience.

#### Scenario design

- **Benchmark Scenario**

This scenario assumes a continuation of the status quo: diesel remains the dominant maritime fuel, and annual growth rates align with historical trends—2.5% for passenger transport, 4.0% for freight, and 5.85% for fisheries—supported by minor technological improvements and limited policy intervention, establishing the reference baseline for measuring carbon footprint reduction.

b) **Proposed Scenario**

Aligned with global climate objectives, this scenario involves a gradual transition to alternative fuels. It includes infrastructure upgrades—port electrification, vessel retrofits—and the integration of renewables. Energy intensity decreases by 1.55% per year, while circular economy measures (e.g., byproduct valorisation) support the transition. Targeted subsidies, including EU-funded schemes, ensure economic feasibility.

#### Analytical framework

The systems-based framework, presented in Fig. 3, evaluates transition trajectories against five interdependent pillars:

1. **Demand Analysis**: Compares economic growth with energy demand.
2. **Transformation Analysis**: Examines scalability of clean technologies such as hydrogen fuel cells and electrification.
3. **Changes in Stock**: Gradual improvement and modernization in infrastructure.
4. **Resource Analysis**: Examines renewable energy resources and fossil fuel reliance.
5. **Environmental Externalities**: Quantifies trade-offs in sustainability, such as emissions and environmental footprint.

## Results

This section presents the simulation results for energy demand and implications for emissions in Albania’s shipping sub-branch and fisheries sector in the baseline and proposed scenarios from 2020 to 2050.

### Model performance and predictor analysis

The MLR model displays strong predictive capability, as indicated by the Multiple R value of 0.93 and an R² of 0.87 reported in Table 2. These statistics demonstrate that 87% of the variance in fishery energy demand is explained by the combined predictors. The adjusted R² of 0.83 accounts for the limited sample size (*n* = 12), enhancing the reliability of the model’s explanatory power. A standard error of 0.04 further reflects the precision of the coefficient estimates, thereby reducing uncertainty in demand projections. Statistical significance is supported by the ANOVA results summarized in Table 3, where the regression sum of squares (0.0679) greatly exceeds the residual sum of squares (0.0098). This yields an F-statistic of 18.41 with a p-value of 0.0006, which rejects the null hypothesis at the 99% confidence level.

.

**Table 2:** Summary of regression statistics

Statistic	Value
Multiple R	0.93
R Square	0.87
Adjusted R Square	0.83
Standard Error	0.04
Observations	12.0

**Table 3:** ANOVA results

Source	df	SS	MS	F	Significance F
Regression	3	0.0679	0.0226	18.4057	0.000598
Residual	8	0.0098	0.0012		
Total	11	0.0777			

However, the regression coefficients (Table 4) indicate that individual predictors are subject to stringent limitations. The intercept (–20.767, *p* = 0.539) suggests a theoretical baseline of energy demand when all predictors are zero; however, its lack of statistical significance diminishes its practical interpretability. Likewise, the coefficient for Year (0.010, *p* = 0.540) reflects a weak temporal trend, while Activity Level (0.016, *p* = 0.210) shows only a marginal influence—neither achieving statistical significance. The GDP variable (0.002, *p* = 0.932) demonstrates a negligible effect, with confidence intervals crossing zero across all predictors.

These findings suggest that while the MLR model captures the broader trends in energy consumption, the insignificance of individual predictors highlights the likely impact of additional, unobserved external factors influencing energy use in Albania’s fishing sector. Expanding the model to include a more comprehensive set of explanatory variables could enhance its predictive power and provide a more robust foundation for sustainable fisheries policy and energy planning.

**Table 4:** Regression coefficients

Predictor Variable	Coefficients	Standard Error	t Stat	*P*-value	Lower 95%	Upper 95%	Lower 95.0%	Upper 95.0%
Intercept	−20.767	32.3543	−0.6418	0.539	−95.376	53.843	−95.376	53.843
Year	0.010	0.0162	0.6406	0.540	−0.027	0.048	−0.027	0.048
Historical Activity Values	0.016	0.0119	1.3644	0.210	−0.011	0.044	−0.011	0.044
GDP	0.002	0.0213	0.0877	0.932	−0.047	0.051	−0.047	0.051

### Impact of IMO 2023 decarbonisation on fisheries emissions

**Fig. 4:**
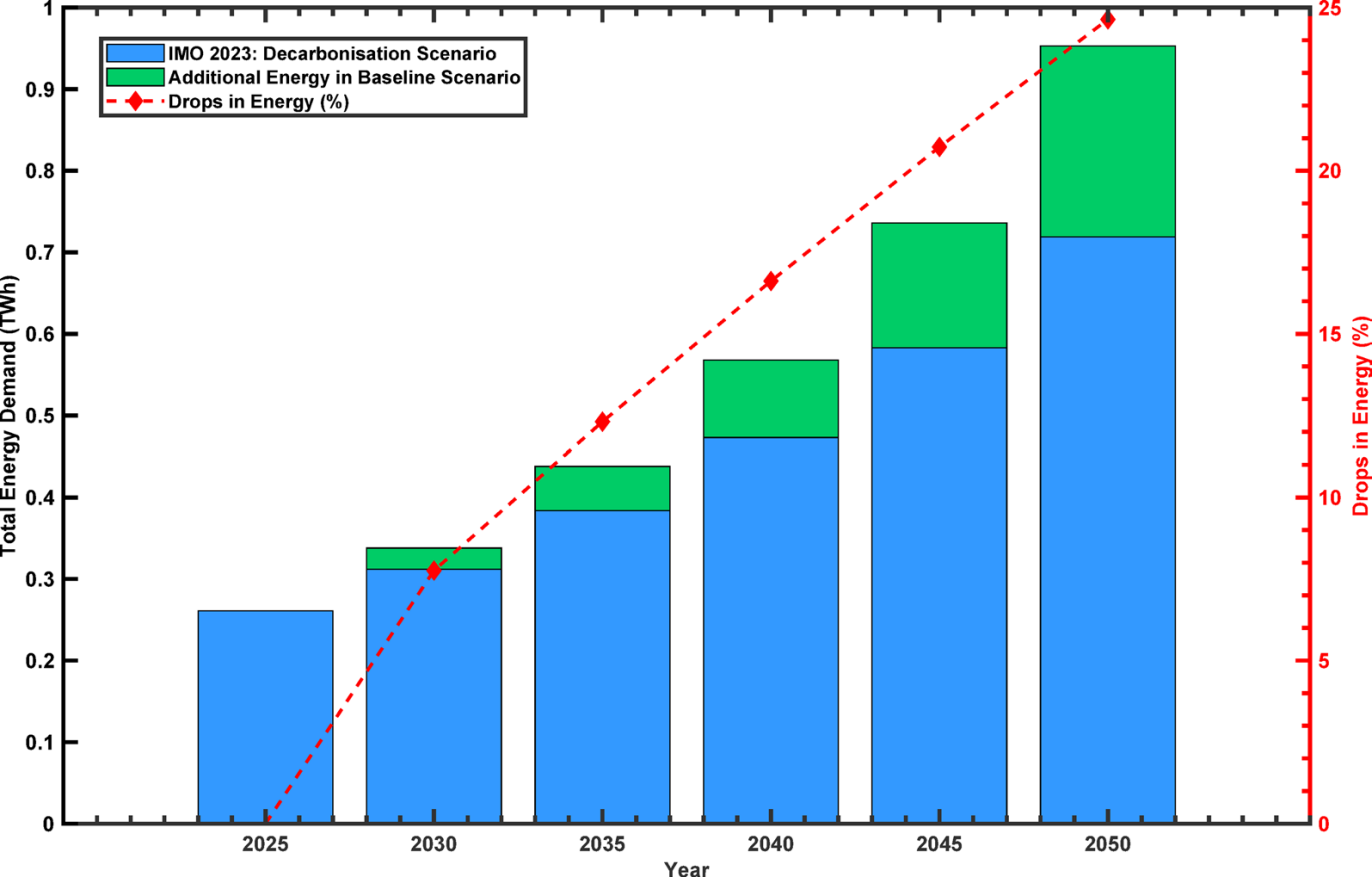
Energy forecast for the fisheries sector across different scenarios

Figure 4 illustrates the total predicted energy demand for the baseline scenario and the proposed scenario, with the avoided energy demand (TWh). In the baseline scenario, the total energy demand continues to grow steadily, increasing to 0.953 TWh by 2050 from 0.261 TWh in 2025. This 265% growth in the 25‑year period is equivalent to an approximate 4.7% growth rate per year. Cumulative energy demand by the year 2050 is 4.087 TWh, pointing towards continuity in traditional energy consumption patterns in the presence of minimal policy effort and no improvements in energy efficiency.

In contrast, the proposed scenario projects a more moderate increase in energy demand, reaching 0.719 TWh by 2050. This constitutes a 175% rise from the 2025 level, equivalent to an average annual growth rate of 3.6%, which is approximately 23% lower than that of the baseline. Cumulative energy demand under this scenario is predicted at 3.517 TWh by 2050, representing a 14% reduction compared to the baseline. This decline is attributed to the introduction of alternative fuels and the implementation of enhanced energy efficiency measures.

Avoided energy demand, representing the difference between the two scenarios, begins at zero in 2025 and increases incrementally throughout the projection period. It reaches 7.76% in 2030, 12.31% in 2035, 16.62% in 2040, 20.73% in 2045, and culminates at 24.64% by 2050. This gradual reduction reflects the combined influence of efficiency improvements and the integration of low‑carbon fuels. The outcome is a forecasted reduction in total energy demand of nearly one‑quarter relative to the baseline by mid‑century, illustrating the potential for meaningful mitigation through sustained policy implementation and technological adaptation.

**Table 5:** Annual fuel share (%) distribution in albania’s fisheries from 2025 to 2050

Energy Source	Year	Crude NGL Feedstock	Electricity	Biodiesel	Hydrogen (H₂)	Other Renewable Energy*
Baseline Scenario	2025	94.9	5.1	-	-	-
	2030	85.6	6.1	8.3	-	-
	2035	82.9	7.1	10.0	-	-
	2040	80.3	8.0	11.7	-	-
	2045	77.6	9.0	13.3	-	-
	2050	75.0	10.0	15.0	-	-
Proposed Scenario	2025	94.9	5.1	-	-	-
	2030	60.0	10.0	10.0	10.0	10.0
	2035	51.5	12.0	12.5	12.0	12.0
	2040	40.0	15.0	15.0	15.0	15.0
	2045	22.5	20.0	17.5	20.0	20.0
	2050	5.0	25.0	20.0	25.0	25.0

*Solar and tidal energy

The forecasted evolution of fuel composition from 2025 to 2050 is presented in Table 5, comparing the Baseline and proposed scenarios. The baseline scenario is marked by continued reliance on conventional fuels, with Crude NGL Feedstock accounting for 94.9% of total consumption in 2025. Its share declines gradually to 75.0% by 2050, representing a decrease of 19.9% points or approximately 21%. Electricity use increases modestly from 5.1% in 2025 to 10.0% in 2050, reflecting a slow trend toward electrification, with an average annual growth rate of approximately 1.96%. Biodiesel emerges in the fuel mix starting in 2030 at 8.3%, increasing to 15.0% by 2050, marking a 6.7%-point rise over two decades. Hydrogen and other alternative fuels are absent in this scenario, indicating minimal uptake of new fuel types under baseline conditions.

The alternative scenario based on IMO 2023 targets anticipates a substantially different pathway. The share of Crude NGL Feedstock falls sharply to 60.0% by 2030 and declines further to just 5.0% by 2050, corresponding to a reduction of 89.9% points relative to 2025. This transformation indicates a near-complete phase-out of conventional fossil fuels.

Electricity consumption in this scenario increases markedly, reaching 25.0% of the total energy mix by 2050, a fivefold rise compared to 2025 and significantly surpassing the baseline’s rate of electrification. Biodiesel also expands, attaining a 20.0% share by 2050, with a more rapid uptake forecast around 2030.

Furthermore, this scenario introduces hydrogen and a suite of renewable fuels, including methanol, ethanol, and ammonia. These alternatives begin entering the mix after 2030, each initially contributing 10.0%, and collectively reaching 25.0% of total energy consumption by mid-century. This transformation in fuel composition signifies a substantial restructuring of the sector’s energy portfolio and aligns the Albanian fishing industry with international climate-mitigation strategies.

**Fig. 5:**
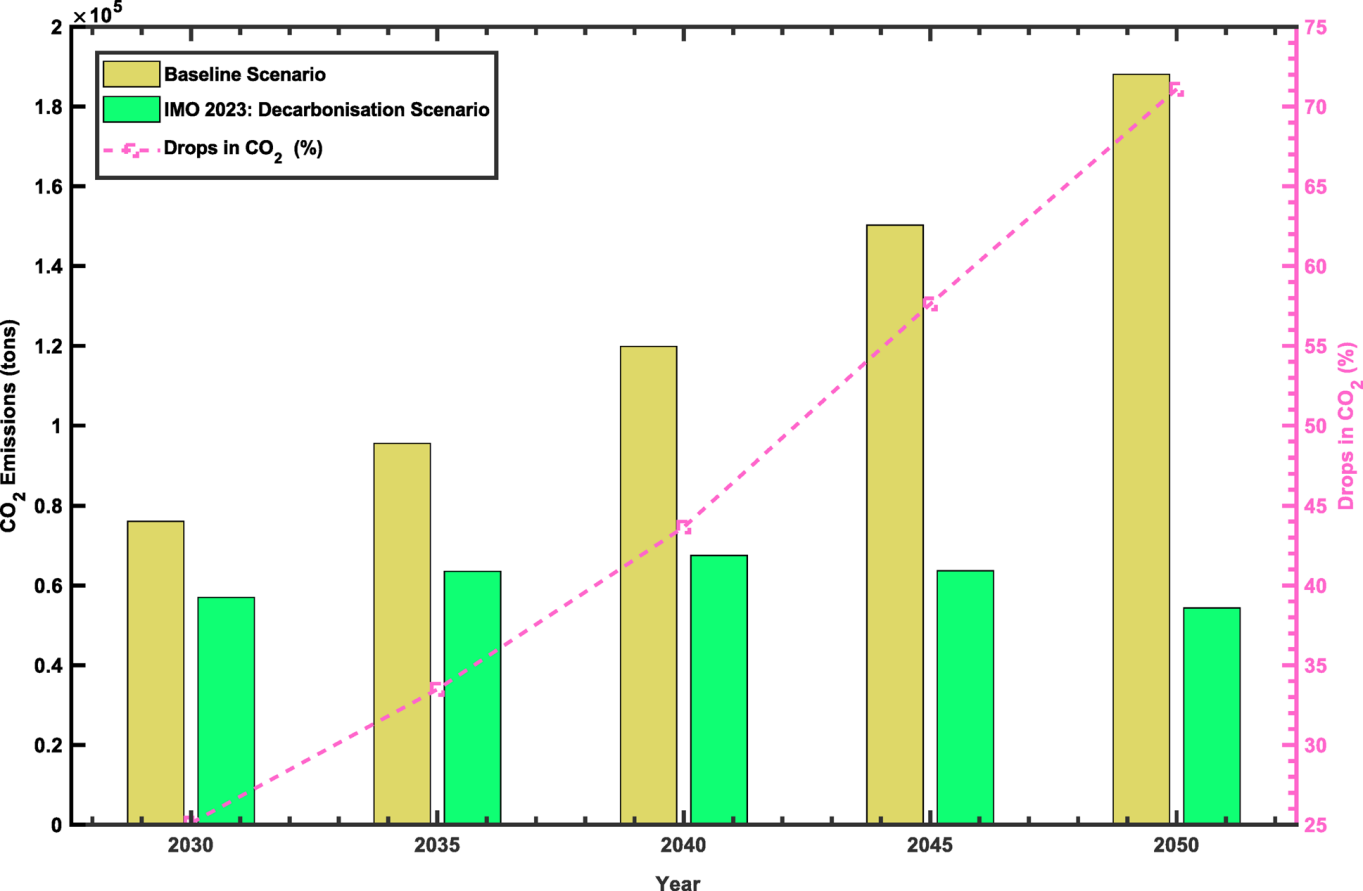
CO₂ Emissions Forecast under Different Scenario

Figure 5 provides a realistic interpretation of the changes in the mix of fuels presented in Table 5, translating shifts in energy composition into quantifiable emissions outcomes. Both scenarios commence from an identical baseline of 60,515 metric tonnes of CO₂ equivalent (CO₂eq) in 2025. By 2030, a divergence becomes evident: the baseline scenario increases to 76,076 metric tonnes of CO₂eq, whereas the IMO 2023-aligned scenario declines to 56,980 metric tonnes of CO₂eq. This corresponds to an absolute reduction of 19,096 metric tonnes of CO₂eq, or 25.1% relative to the Baseline, reflecting the 34.9%-point decrease in Crude NGL Feedstock consumption over the same period.

The disparity in emissions expands over time. In 2040, emissions under the baseline scenario reach 119,880 metric tonnes of CO₂eq, compared to 67,541 metric tonnes of CO₂eq under the proposed pathway, resulting in 52,339 metric tonnes of CO₂eq avoided, equivalent to a 43.7% reduction. The most substantial difference appears in 2050, with baseline scenario emissions peaking at 188,091 metric tonnes of CO₂eq, while the Alternative Scenario achieves a considerable decline to 54,382 metric tonnes of CO₂eq. This contrast amounts to 133,709 metric tonnes of CO₂eq in avoided emissions, representing a 71.1% decrease compared to the Baseline projection.

Overall, emissions from the fishing sector are forecast to increase by more than threefold throughout the analysis period in the absence of mitigation efforts. The application of carbon removal strategies has the potential to achieve a reduction of 28.9%, relative to the baseline estimate, by 2050.

### Navigation transport sub-branch forecast

**Fig. 6:**
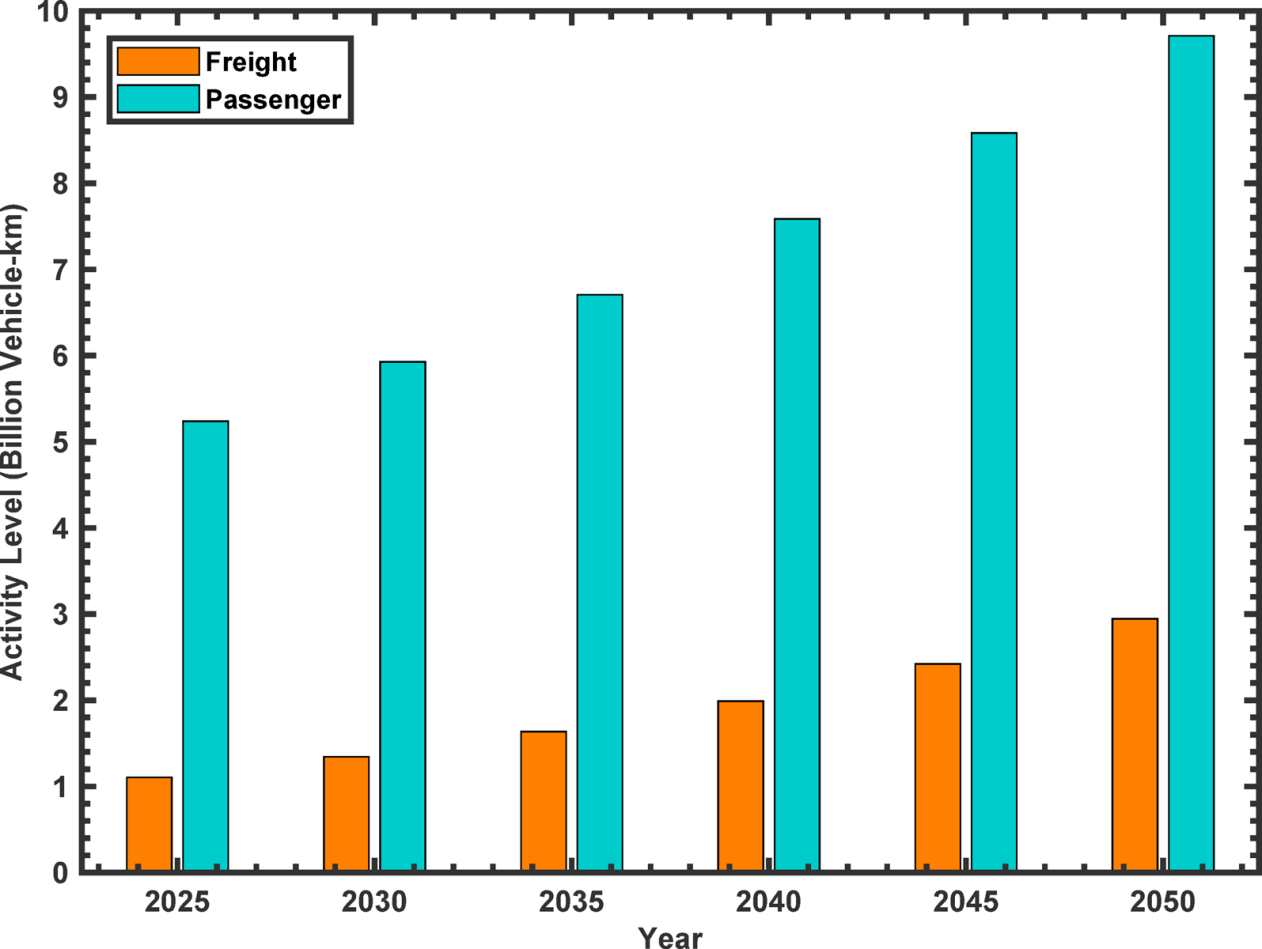
Forecasted activity levels in the transport sector, covering passenger and freight sub-sectors

In this subsection, future transport trends for navigation, passenger, and freight transport are forecast for the period 2025 to 2050. First, a comparison of the levels of passenger and freight activity in billion vehicle-kilometers is presented in Fig. 6. Based on the data, there is a sustained increase in passenger and freight transport over the 25-year period. Passenger activity starts at 5.238 billion vehicle-km in 2025 and has a sustained increasing pattern up to 9.711 billion vehicle-km in 2050. This represents a rise of approximately 85.3%, reflecting growing demand for passenger transport services. On the other hand, freight activity begins with a lower base of 1.105 billion vehicle-km in 2025 but follows a faster growth trajectory to reach 2.945 billion vehicle-km in 2050. This rapid growth of about 166.5% points to the growing role of freight transport.

**Table 6:** Forecasted fuel mix evolution in albania’s navigation transport sector under baseline and proposed scenarios (2025–2050)

Energy Source	Year	Crude NGL Feedstock	Electricity	Biodiesel	Hydrogen (H₂)	Other Renewable Energy*
Baseline scenario	2025	100.0	-	-	-	-
	2030	90.0	10.0	-	-	-
	2035	90.0	10.0	-	-	-
	2040	90.0	10.0	-	-	-
	2045	90.0	10.0	-	-	-
	2050	90.0	10.0	-	-	-
Proposed scenario	2025	100.0	-	-	-	-
	2030	64.4	10.0	10.0	10.0	2.8
	2035	51.4	12.0	15.0	15.0	3.3
	2040	37.2	15.0	20.0	20.0	3.9
	2045	21.1	20.0	25.0	25.0	4.4
	2050	5.0	25.0	30.0	30.0	5.0

*Solar and tidal energy

Secondly, an analysis of the fuel distribution within Albania’s navigation sub-branch provides insight into how energy sources evolve under different scenarios. Table 6 presents a comparative assessment of fuel use under the proposed framework. In the baseline scenario, reliance on Crude NGL Feedstock remains unaltered, with a 90.0% contribution from 2030 to 2050, and only a 10.0% contribution from electricity added in 2030. Conversely, in the IMO 2023 pathway, the contribution of Crude NGL Feedstock plummets sharply from 100.0% in 2025 to as low as 5.0% in 2050, with a dramatic 95.0% decline. This decline is counterbalanced by the progressive rise in electricity, biodiesel, hydrogen (H₂), and other renewables. By 2050, the scenario anticipates the same contribution of 30.0% for hydrogen and biodiesel, 25.0% for electricity, and 5.0% for other renewables. The point of inflection occurs in the 2030–2040 period, marking the critical transition towards decarbonization when the cumulative contribution of alternative fuels crosses the 50% threshold for the first time. The growth trajectory of hydrogen uptake is particularly remarkable, increasing from 10.0% in 2030 to 30.0% in 2050, cementing hydrogen as one of the key vectors of emissions reductions. Similarly, biodiesel consumption follows the same trajectory, reaching 30.0% in 2050, signaling a complementary role in the decarbonization mix. The progressive rise in electricity to 25.0% in 2050 signals the increasing viability of maritime electrification, most likely supported by upgrades to battery storage and port charging equipment. Conversely, the contribution of other renewables is modest but nearly doubles, rising from 2.8% in 2030 to 5.0% in 2050, supporting the energy-diversification trend.

**Fig. 7:**
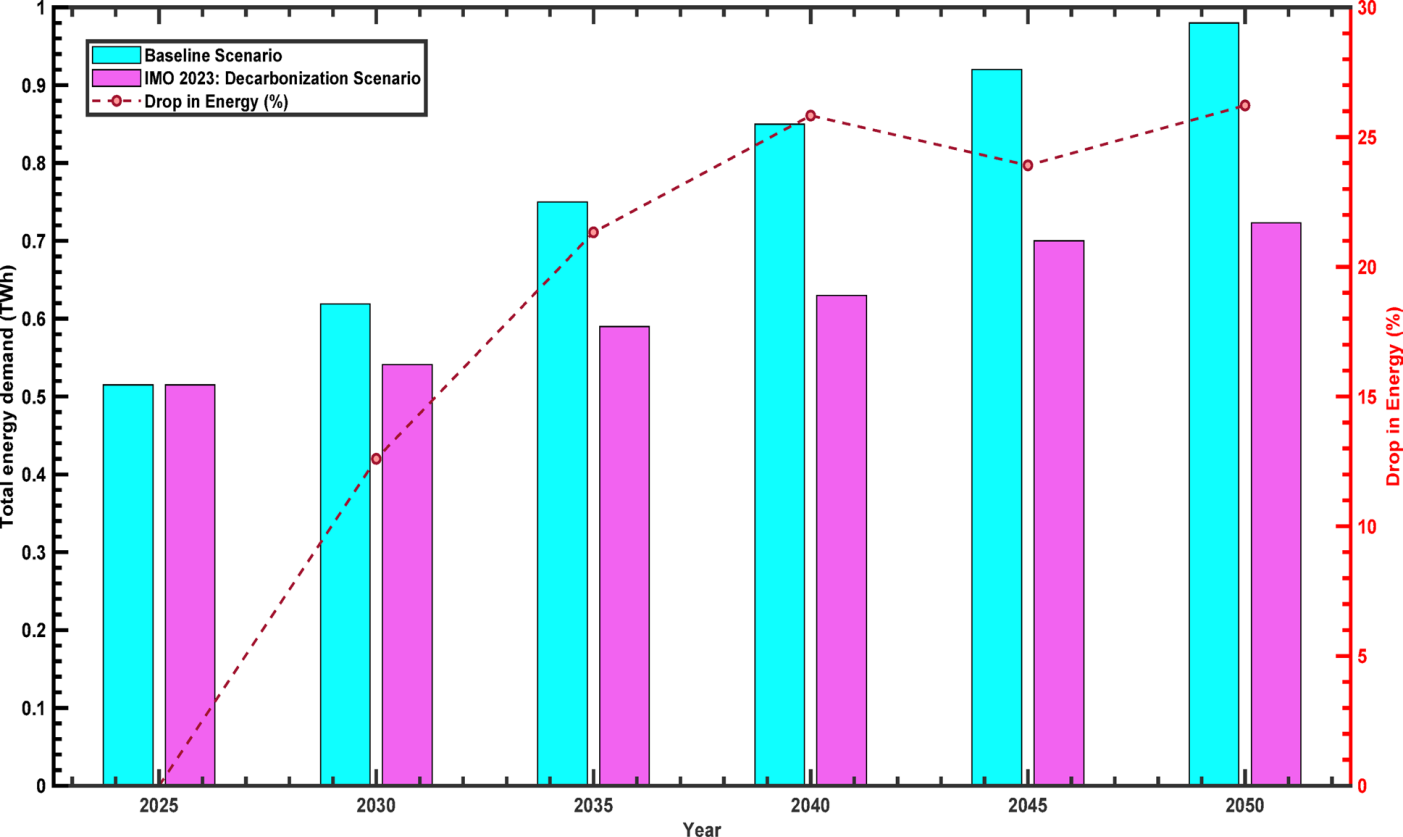
Energy demand (TWh) for the case of baseline and IMO 2023: Decarbonisation scenario

Thirdly, the analysis of total energy demand reveals the broader implications of these fuel shifts. Figure 7 compares total energy demand for Albania’s navigation sub-branch under the two scenarios from 2025 to 2050. Under the Baseline scenario, total energy demand increases steadily from 0.515 TWh in 2025 to 0.980 TWh in 2050, reflecting the overall growth of approximately 90.3% over the period. In contrast, the proposed scenario exhibits a more restrained growth trajectory, with demand reaching 0.723 TWh in 2050, corresponding to an increase of 40.4%.

The percentage of energy savings follows a gradually rising trend, peaking at around 27% in 2040 and stabilizing slightly at 26.2% by 2050. The widening gap in energy demand between the two scenarios over time reflects the growing influence of decarbonization strategies. In 2050, energy consumption under the Baseline scenario exceeds that of the proposed pathway by 35.6%, underscoring the cumulative effect of sustained mitigation efforts.

**Fig. 8:**
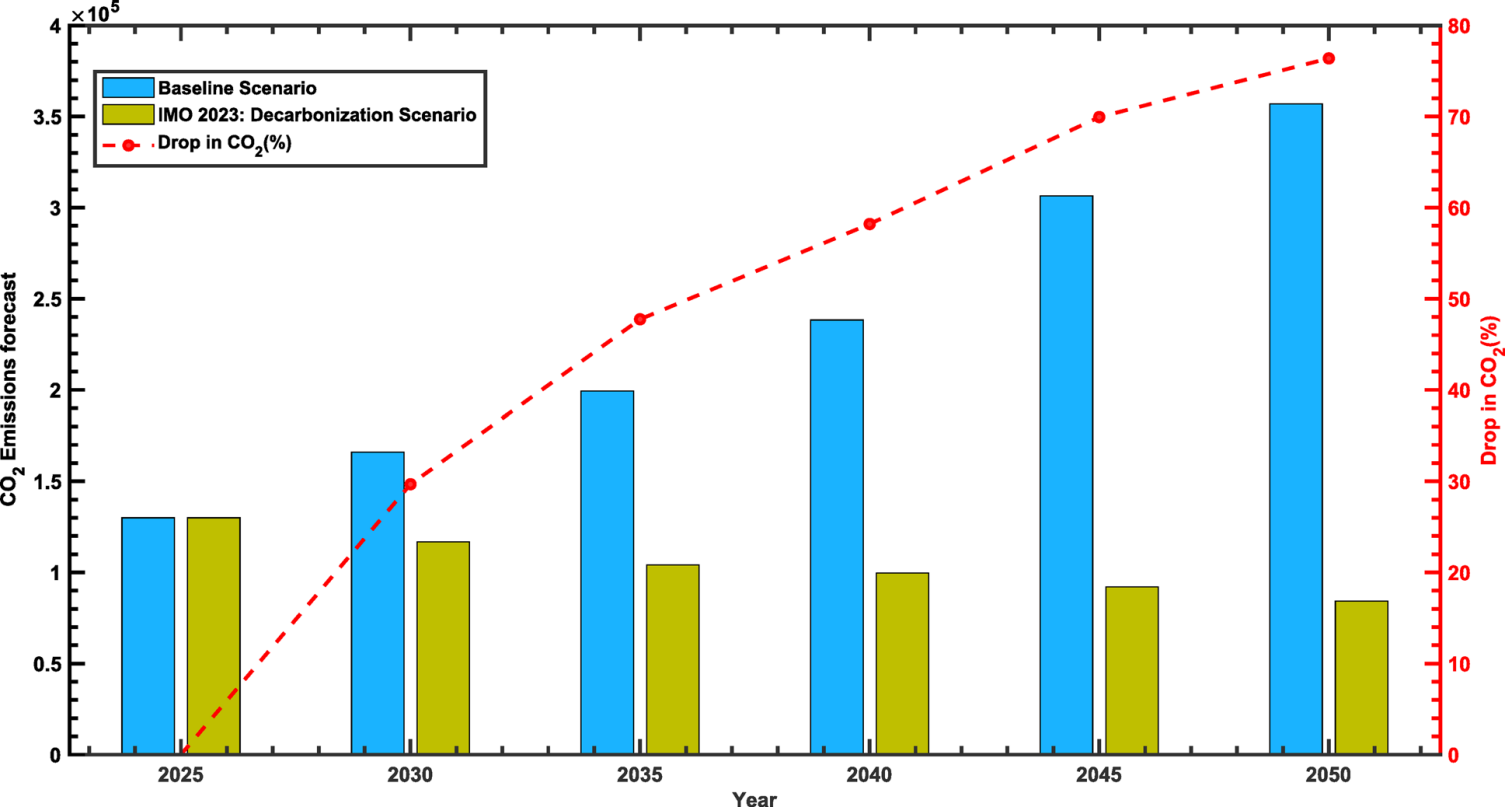
Comparative Analysis of CO₂ Emissions Under Baseline and proposed Scenarios

Finally, the forecasted CO₂ emissions from Albania’s navigation sub-sector reflect the influence of evolving energy trends, as depicted in Fig. 8. Under the baseline scenario, emissions increase from 129,990 tons in 2025 to 357,007 tons in 2050, representing a 174.6% rise over the period. On the other hand, the IMO 2023-aligned pathway exhibits a consistent decline in emissions, reaching 84,313 tons by 2050, an absolute reduction of 35.1%. After 2030, the difference between the two scenarios widens significantly, with emissions in the mitigation pathway 47.75% lower by 2035 and 76.38% lower by 2050.

The trajectory of emissions reductions (Fig. 8) also reveals a marked acceleration beyond 2030. Reductions surpass 50% by 2040 and exceed 70% by 2045. By 2050, emissions under the IMO 2023 pathway account for only 23.6% of those forecasted in the baseline scenario, underscoring the transformative potential of sustainable energy-transition measures. Realizing these outcomes will depend on sustained investments in alternative-fuel technologies and the rigorous implementation of regulatory frameworks designed to ensure significant emissions mitigation within the maritime sector.

## Discussion

The results of this study offer meaningful insights into the evolving patterns of energy demand and greenhouse gas (GHG) emissions from Albania’s fisheries and maritime transport sectors under both the baseline and proposed scenarios. The analysis integrates the LEAP system to simulate future energy trajectories and estimate associated GHG emissions under supply and policy uncertainties. Furthermore, MLR models were employed to estimate long-term trends in activity levels for freight and passenger transport within the maritime domain.

The MLR model used in the analysis exhibits strong predictive accuracy, reflected by a high coefficient of determination (R² = 0.87). However, the low statistical significance of individual predictors suggests that the model does not capture several influential external factors. This highlights a notable constraint in the current framework and signals the need for more comprehensive modeling approaches [32]. Further improvements in predictive reliability are likely to be achieved by incorporating additional variables as well as exploring machine learning techniques for capturing complex interactions [33–39].

Under the baseline scenario, projections indicate continued reliance on crude oil and natural gas liquids (NGLs), accompanied by minimal incorporation of biofuels or electrified propulsion systems. This outcome reflects prevailing global patterns in the fisheries sector, where fossil fuel use remains predominant despite stated decarbonization targets [40]. The proposed scenario, in contrast, exhibits an observable shift in the energy mix, characterized by reduced fossil fuel dependence and increased utilization of alternative energy carriers, including hydrogen. This development corresponds to global assessments that identify hydrogen as a key enabler in maritime energy transition, supported by declining production costs and favorable regulatory frameworks [41]. Regional initiatives in countries such as Greece and Spain further substantiate this trend. EU-funded projects, including H2-SEAS and HY2FISH, have demonstrated the feasibility of hydrogen integration by retrofitting small fleets with hybrid systems, resulting in fuel savings of approximately 30–40% during pilot implementation phases [42].

A forecasted reduction of 71.1% in GHG emissions by 2050 under the proposed scenario is consistent with international maritime decarbonization targets. Evidence from the literature supports the technological feasibility of such reductions through an integrated strategy involving fuel switching, electrification, and enhancements in operational energy efficiency. The forecasted total emissions reduction potential of 1.473 MtCO₂eq underscores the fisheries and maritime transport sub-sectors’ capacity to deliver significant contributions to national climate goals when contingent upon targeted policy interventions and coordinated planning. However, the pathway to implementation remains constrained by systemic, financial, and regulatory barriers. Overcoming these challenges will require synchronized public-sector funding, regulatory reform, and international collaboration. Leveraging EU financing mechanisms can play a critical role in facilitating infrastructure upgrades and demonstration projects.

In relation to maritime transport, baseline projections indicate sustained growth in energy consumption and emissions. In contrast, the proposed scenario indicates a substantial reduction in emissions, consistent with international research findings regarding emissions mitigation in global shipping through alternative fuels and electrified systems [43]. These outcomes underscore the potential benefits of integrating maritime carbon neutrality objectives into broader transportation planning strategies.

In summary, this study enriches the understanding of energy trajectories and emissions in Albania’s maritime and fisheries sectors, while offering actionable guidance for policymakers. The findings highlight the importance of integrated strategic planning that draws upon regional best practices, aligns with European financing instruments, and is underpinned by institutional coherence and coordinated multilevel governance structures.

## Conclusion

This study has assessed the long-term energy demand and emissions pathways of Albania’s fisheries and navigation sectors under a baseline trajectory and an alternative scenario aligned with the IMO 2023 decarbonization objectives. The adopted methodological framework relied on quantitative modeling using the LEAP system and MLR analysis to simulate energy demand, fuel transitions, and emissions outputs.

The analysis reveals a marked divergence between the baseline and alternative scenarios. Under baseline conditions, energy demand in the fisheries sector is projected to increase from 0.261 TWh in 2025 to 0.953 TWh by 2050, highlighting continued reliance on fossil fuels. Consequently, related emissions are expected to rise significantly, exceeding three times the 2025 levels by mid-century. In contrast, the alternative scenario projects a more moderate increase in energy use and a cumulative emissions reduction of 71.1% by 2050. This is primarily due to the phased replacement of crude oil–based fuels with biodiesel, electricity, hydrogen, and other clean energy alternatives.

In parallel, the navigation sub-sector shows similar trends. Under the baseline scenario, energy demand is predicted to increase by 90.3%, accompanied by a 174.6% rise in greenhouse gas emissions. However, the alternative scenario foresees a 76.38% reduction in emissions by 2050 relative to the baseline scenario. Fossil fuel dependence falls from full reliance in 2025 to just 5% in 2050, supplanted by electricity, biodiesel, hydrogen, and other renewable fuels. These shifts are also associated with a noticeable decline in total energy demand, which levels off at 0.723 TWh in 2050 under the alternative scenario, compared to 0.980 TWh under the baseline scenario.

Further projections indicate that hydrogen adoption will increase from 10% in 2030 to 30% by 2050, with biodiesel use showing a comparable trend. Electricity is expected to reach 25% of the navigation energy mix by 2050, reflecting the increasing viability of electrification, driven by advances in battery storage and charging infrastructure. The projected mitigation of 1.473 MtCO₂eq underscores the potential of the maritime and fisheries sectors to contribute meaningfully to national climate targets when supported by appropriate regulatory frameworks and strategic investments.

Overall, emissions mitigation in both sectors under the proposed scenario is consistent with broader transport sector objectives and illustrates the feasibility of aligning national maritime energy systems with climate neutrality targets. Integrating national strategies with regional financing mechanisms can strengthen implementation efforts and enhance overall effectiveness.

Future work should prioritize the refinement of predictive models by incorporating a wider range of economic, environmental, and operational parameters. It is also essential to assess the viability of emerging technologies under localized economic and regulatory contexts. In addition, further research is warranted to examine the institutional capacity and financing mechanisms required to scale up decarbonization efforts, particularly in areas such as port infrastructure and fuel supply chains.

## Data Availability

All data are provided in the paper’s content.
